# Ischemic stroke and dose adjustment of oral Factor Xa inhibitors in patients with atrial fibrillation

**DOI:** 10.1007/s00415-020-09795-3

**Published:** 2020-03-23

**Authors:** Svenja Stoll, Kosmas Macha, Armin Marsch, Stefan T. Gerner, Gabriela Siedler, Kilian Fröhlich, Bastian Volbers, Erwin F. Strasser, Stefan Schwab, Bernd Kallmünzer

**Affiliations:** 1 Department of Neurology, University Hospital Erlangen, Friedrich-Alexander-University Erlangen-Nuremberg (FAU), Schwabachanlage 6, 91054 Erlangen, Germany; 2 Department of Transfusion Medicine and Haemostaseology, University Hospital Erlangen, Friedrich-Alexander-University Erlangen-Nuremberg (FAU), Erlangen, Germany

**Keywords:** Direct oral anticoagulants, Stroke, Plasma levels, Atrial fibrillation, Dose reduction

## Abstract

**Background:**

Oral Factor Xa inhibitors for the prevention of stroke in atrial fibrillation require dose adjustment based on certain clinical criteria, but the off-label use of the reduced doses is common.

**Methods:**

Data from an observational registry including patients admitted with acute cerebral ischemia while taking oral Factor Xa inhibitors for atrial fibrillation between April 2016 and December 2018 were investigated. The dose regimen of the Xa inhibitor was classified as “appropriate”, “underdosed” and “overdosed” in conformity with the European Medicines Agency labelling. The effect of underdosing on the functional factor Xa plasma level on admission, the clinical stroke severity and the functional outcome after 3 months were investigated.

**Results:**

254 patients with cerebral ischemia while on Factor Xa inhibitors were included. The dose regimen of the Factor Xa inhibitor was appropriate in 166 patients (65%), underdosed in 67 patients (26%) and overdosed in 21 patients (8%). Underdosing was associated with female sex, diabetes mellitus and higher CHA_2_DS_2_–Vasc scores. Underdosing independently predicted lower anti-Xa plasma levels on admission [median 69.4 ng/ml (IQR 0.0–121.6) vs. 129.2 ng/ml (65.5–207.2); *p* < 0.001], was associated with higher NIHSS scores on admission [median 5 (IQR 1–10) vs. 3 (1–7); *p* = 0.041] and worse functional outcome after 3 months (favorable outcome 26.9% vs. 46.9%; *p* = 0.025).

**Conclusion:**

One in three patients with ischemic stroke during treatment with oral Xa inhibitors used inappropriate dose regimens. Underdosing was associated with lower functional plasma levels, higher clinical stroke severity and worse functional outcome.

## Background

Atrial fibrillation (AF) is the leading cause of cardioembolic complications causing up to 25% of acute ischemic strokes [1–3]. AF is associated with an elevated risk of in-hospital mortality among patients with ischemic stroke and even among those with a cardioembolic stroke subtype [4]. Oral anticoagulation (OAC) with vitamin K antagonists (VKA) or direct oral anticoagulants (DOAC) is the standard of care for primary and secondary prevention in patients with non-valvular AF [1, 5]. Compared to VKA, DOACs provide a superior benefit–risk profile [6–10], mainly driven by a lower risk of intracranial bleeding complications [11, 12]. The dose regimens of the three currently available oral Xa inhibitors warrant adjustment to lower daily doses in certain clinical conditions, while the criteria for the reduction were defined substance-specifically and include age, different degrees of chronic renal failure, body weight and the risk of drug interactions [1, 13]. However, data from real word cohorts reported a significant proportion of patients to be using an inappropriate low dose of these substances. This off-label use was associated with an elevated risk of hospitalization and death [11, 14]. In the present study we explored the rate of Factor Xa inhibitor underdosing among patients with acute cerebral ischemia, its effects on Xa inhibitor plasma levels, stroke severity as well as the functional outcome after 3 months.

## Methods

### Study design

The protocol was approved by the ethics committee of the Faculty of Medicine, University of Erlangen-Nuremberg, Germany. The anonymized data that support the findings of this study are available from the corresponding author on request. Consecutive patients taking oral Factor Xa inhibitors for atrial fibrillation at the time of stroke onset between April 2016 and December 2018 were identified from our institutional registry “Erlangen Registry of Patients on Oral Anticoagulation (ER-NOAC)”, including clinical, demographic and laboratory data [15]. Patients received acute care in a university neurologic stroke unit or dedicated neurointensive care unit following international recommendations [15].

### EMA-labelling

The Xa inhibitor doses were classified as “appropriate”, “underdosed” or “overdosed” in consistency with the European Medicines Agency (EMA). A synopsis of the dose regimens and the relevant criteria are provided with Table 1. The classification was based on the clinical and laboratory findings at the time of hospital admission.

**Table 1:** Synopsis of dose regimens and criteria for dose adjustment according to the European Medicines Agency labelling [1]

	Apixaban	Rivaroxaban	Edoxaban
Regular dose	5 mg b.i.d.	20 mg q.d.	60 mg q.d.
Reduced dose	2.5 mg b.i.d.	15 mg q.d.	30 mg q.d.
Dose reduction	At least two of the following characteristics Age ≥ 80 years Body weight ≤ 60 kg Serum creatinine ≥ 1.5 mg/dl Or: creatinine clearance (Cr-Cl) 15–29 ml/min	Cr-Cl 15–49 ml/min	Cr-Cl 15–50 ml/min Body weight ≤ 60 kg Intake of p-glycoprotein inhibitor: ciclosporin, erythromycin, dronedarone, ketoconazole

*Cr-CL* creatinine clearance, *q.d.* quaque die/once a day, *b.i.d.* bis in die/twice a day

### Laboratory diagnostics

The standard of care included a renal function test and calculation of the creatinine-clearance using the Cockcroft–Gault formula [16]. In addition to local standards, the specific plasma level of the anticoagulant on admission was measured in patients with acute ischemic stroke, using anti-Xa-based chromogenic assays (STA-Liquid Anti-Xa, Diagnostica STAGO S.A.S., France) with anticoagulant-specific calibration (Diagnostica STAGO S.A.S., France). Plasma levels below the detection limit (20 ng/ml) were set to zero for measurement reasons.

### Endpoints

The primary endpoints were the functional DOAC plasma level and the clinical stroke severity on admission, measured on the National Institutes of Health Stroke Scale (NIHSS). The secondary endpoint was the functional outcome on the modified Rankin Scale (mRS, scores 0–6) after 3 months.

### Statistical analyses

Statistical analyses were performed using the SPSS software package (IBM SPSS Statistics 21). Data were tested for normality of distribution using the Shapiro–Wilk and Kolmogorov–Smirnov tests and are presented as absolute/relative numbers or median/interquartile range (IQR). Patients with inappropriate dose reduction on admission were compared to those with approved regimens using the non-parametric Mann–Whitney *U* test, the Pearson’s chi-square test or Fisher’s exact test as indicated. Statistical significance level was set at *p* values < 0.05. A univariate regression model was calculated to identify factors that predict the specific factor Xa plasma level.

## Results

Two hundred fifty-four patients with acute cerebral ischemia while on treatment with oral Factor Xa inhibitors for atrial fibrillation were included. In 166 patients (64.8%) the dose regimen of the factor Xa inhibitor prior to stroke onset followed the criteria of the EMA-labelling, 67 patients (26.2%) were classified as underdosed and 21 patients (8.2%) as overdosed. The rate of inappropriate dose regimens did not differ significantly between the three substances (Table 2).

**Table 2:** Rate of off-label use at the time of stroke onset for the three oral factor Xa inhibitors

	Appropriate dose	Underdosed	Overdosed
Apixaban, *n* = 162	104 (64.2%)	46 (28.4%)	11 (6.8%)
Rivaroxaban, *n* = 62	40 (64.5%)	16 (25.8%)	5 (8.1%)
Edoxaban, *n* = 32	22 (68.8%)	5 (15.6%)	5 (15.6%)

Table 3 shows the demographic and clinical characteristics of patients with dose regimens classified as appropriate or underdosed. Underdosing was associated with female sex, diabetes mellitus and CHA_2_DS_2_–Vasc score.

**Table 3:** Baseline characteristics of patients using the regular dose or the inappropriate low dose of the factor Xa inhibitor at the time of stroke onset

	Regular dose on admission (*n* = 166)	Underdosed on admission (*n* = 67)	*p* value
Age, median (IQR)	80 (75–85)	81 (77–86)	0.155
Female sex, *n* (%)	78 (47.0)	43 (64.2)	**0.017**
Body weight in kg, median (IQR)	78.0 (69.0–85.0)	74.0 (65.0–85.0)	0.247
Arterial hypertension, *n* (%)	156 (94.0)	62 (92.5)	0.288
Diabetes mellitus, *n* (%)	51 (30.9)	33 (49.3)	**0.008**
Hypercholesterolaemia, *n* (%)	135 (82.8)	53 (80.3)	0.652
Normal renal function, *n* (%)	101 (60.8)	33 (49.3)	0.105
Prior stroke, *n* (%)	80 (48.5)	26 (38.8)	0.314
CHA_2_DS_2_–VASc score, median (IQR)	6.0 (5.0–7.0)	7.0 (6.0–8.0)	**0.008**
Pre-mRS, median (IQR)	1.0 (0–3)	2.0 (0–3)	0.071
Intake of edoxaban on admission, *n* (%)	22 (13.3)	5 (7.5)	0.262
Intake of apixaban on admission, *n* (%)	104 (62.7)	46 (68.7)	0.386
Intake of rivaroxaban on admission, *n* (%)	40 (24.1)	16 (23.9)	0.972
Thrombectomy, *n* (%)	16 (9.6)	9 (13.4)	0.397
Thrombolysis, *n* (%)	12 (7.2)	4 (6.0)	0.492

*IQR* interquartile range, normal renal function is defined as glomerular filtration rate > 60 ml/min/1.73 m^2^, *kg* kilogram, *(pre-)mRS* (pre-)modified Rankin Scale. Numbers in bold indicate statistical significance (*p* < 0.05)

Specific factor Xa inhibitor plasma levels were available for 215 subjects (84.0%) on admission. Patients with inappropriate dose reduction prior to admission had significantly lower plasma levels [median 69.4 ng/ml (IQR 0.0–121.6) versus 129.2 ng/ml (65.5–207.2); *p* < 0.001].

Table 4 shows the results from the logistic regression analysis of specific factor Xa plasma levels on admission. Underdosing was associated with lower substance specific plasma levels (*β* − 0.261, *p* < 0.001).

**Table 4:** Logistic regression model for the factor Xa plasma level on admission

Variable	Regression *β* coefficient	*p* value
Age	0.110	0.123
Female sex	− 0.066	0.357
CHA_2_DS_2_–VASc	0.041	0.572
Normal renal function	− 0.047	0.511
Diabetes mellitus	0.019	0.792
Body weight	− 0.083	0.252
Pre-mRS	− 0.034	0.633
Underdosed factor Xa inhibitor	− 0.261	< 0.001

**Fig. 1:**
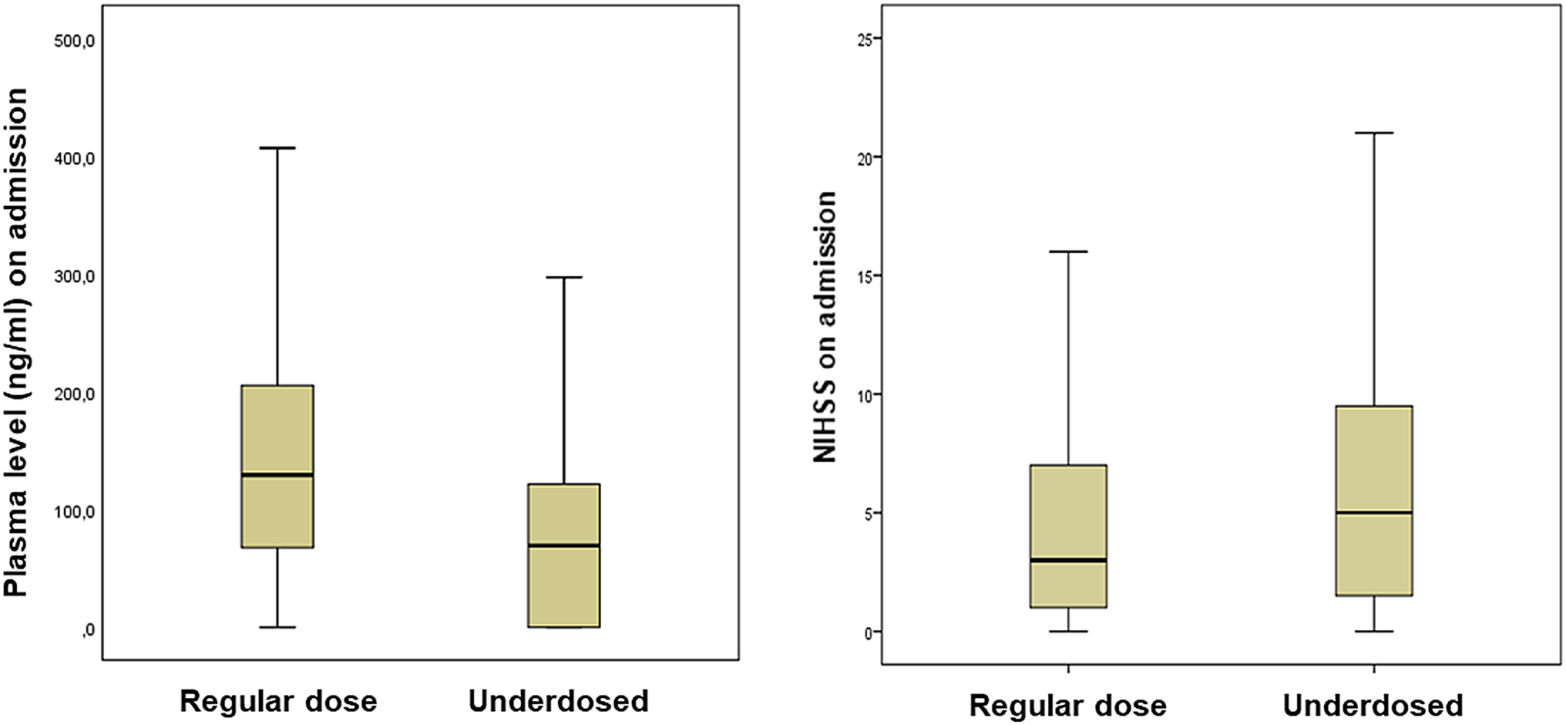
Effect of underdosing on specific plasma levels and initial stroke severity. Left: median Xa inhibitor plasma level (ng/ml) on admission: 69.4 ng/ml (IQR 0.0–121.6) versus 129.2 ng/ml (65.5–207.2); *p* < 0.001. Right: median National Institutes of Health Stroke Scale (NIHSS) score on admission: 3 (IQR 1–7) versus 5 (1–10); *p* = 0.041

The clinical stroke severity among patients with an inappropriate dose regimen at the time of stroke onset was higher than among patients with approved doses, the scores based on the NIHSS on admission were higher [median 5 (IQR 1–10) versus 3 [1–7]; *p* = 0.041; Fig. 1].

Three months after acute cerebral ischemia, the rate of recurrent cardioembolic events was not different (2.3%), but the rate of favorable functional outcome was significantly less common among patients who were underdosed on admission [18 (30.0%) versus 69 (46.9%); *p* = 0.025; Table 5].

**Table 5:** Primary and secondary outcome in patients according to the dose regimen prior to the stroke

	Regular dose on admission (*n* = 166)	Underdosed on admission (*n* = 67)	*p* value
NIHSS on admission, median (IQR)	3.0 (1.0–7.0)	5.0 (1.0–10.0)	**0.041**
Plasma level on admission in ng/ml, median (IQR)	129.2 (65.5–207.2)	69.4 (0–121.6)	**< 0.001**
Stroke, *n* (%)	113 (68.1)	50 (74.6)	0.323
Large vessel occlusion, *n* (%)	15 (20.8)	7 (30.4)	0.342
Mortality in-hospital, *n* (%)	8 (4.9)	4 (6.1)	0.476
NIHSS at discharge, median (IQR)	1.0 (0–4.0)	2.0 (0–5.0)	0.129
Favorable outcome (mRS after 3 months 0–1 or idem), *n* (%)	69 (46.9)	18 (30)	**0.025**
Favorable outcome (mRS after 3 months 0–2 or idem), *n* (%)	81 (55.1)	23 (38.3)	**0.029**
Mortality within 3 months, *n* (%)	27 (18.1)	15 (25.0)	0.262

*NIHSS* National Institutes of Health Stroke Scale. Numbers in bold indicate statistical significance (*p* < 0.05)

## Discussion

There are three major findings from this study: (1) one in three patients with stroke and factor Xa inhibitors for atrial fibrillation used an inappropriate dose regimen of the anticoagulant at the time of stroke onset; (2) underdosing lead to significantly lower plasma levels on admission and (3) underdosed patients showed significantly higher NIHSS scores on admission and a worse functional outcome after 3 months.

In earlier studies, the off-label use of anticoagulants with inappropriate dose regimens was a frequent and potentially harmful phenomenon in the clinical care for patients with atrial fibrillation: Steinberg et al. reported underdosing in almost 10% of patients taking DOACs mainly for primary prevention [14]. In this cohort, the off-label use was associated with a high risk of hospitalization and death. In our study, the rate of inappropriate dose regimens among patients with acute stroke was as high as 34%. However, due to a relevant selection bias, this observation does not allow any conclusion on stroke etiology and underdosing might simply constitute a surrogate for higher stroke risk found in other reasons.

Arboix et al. reported that the recurrent embolization in the first days after stroke is an important predictor of the in-hospital mortality [17]. For early secondary prevention it might, therefore, be of particular relevance to perform a critical review of the DOAC dose that was used prior to the stroke with consecutive alignment to an approved regimen in cases of under- or overdosing. Following this review and alignment, there was no significant difference in the rate of recurrent cardioembolic events within the following 3 months.

In concurrence to the literature, our data indicate that inappropriate dose reduction is particularly common among women and patients with higher CHA_2_DS_2_–Vasc scores [14]. Other studies reported repetitive falls and dementia as additional predictors of underdosing [18–20]. However, large clinical trials clearly demonstrated a preserved benefit–risk profile of the substances in the regular dose and international treatment guidelines do not recommend general dose reduction for these subgroups [21]. Perhaps more detailed information and educative measures among patients and health care professionals could help to improve care by confining the use of inappropriate doses [22].

The degree of renal elimination is different among the three factor Xa inhibitors and the criteria for dose adjustment do not represent these differences as a whole, while the classification into appropriate and inappropriate doses was based on the official EMA label. Especially the presence of diabetes mellitus could cause relevant bias to this classification. Treatment with DOACs does not require hemostaseologic monitoring by routine. However, chromogenic factor Xa assays with substance specific calibration correlate reliably with the antithrombotic activity and allow the measurement of a functional factor Xa inhibitor plasma level in case of an emergency [23–25]. The test was included into the standard of care for patients with acute ischemic stroke at our department, thereby supporting the decisions of recanalization therapies including intravenous thrombolysis [26]. It seems self-evident, that reduced dose regimens would result in lower plasma levels among patients with acute stroke. Consistently, previous research reported an inverse correlation between the antithrombotic activities at the time of stroke onset with the initial stroke severity among both, patients using vitamin K antagonists and DOACs [27]. Notably, in our study the dose regimen at the time of stroke onset was still associated with the functional outcome 3 months later. A higher rate of spontaneous vessel recanalization, changes in the consistency and size of intracardial thrombi or a reduced risk of early stroke recurrence are suggested as possible mechanisms [14]: in the ENGAGE AF-TIMI 48 trial the stroke risk was higher in patients with reduced doses of edoxaban [28], and the Randomized Evaluation of Long-Term Anticoagulation Therapy (RE-LY) demonstrated an inverse correlation between dabigatran plasma levels and probability of an ischemic stroke [29].

The single center design and the rather small number of patients are certainly relevant limitations to our study. The classification of the dose regimen as “inappropriate” depended on the serum creatinine on admission. The reliability of this parameter to assess the renal function is limited, as various factors contribute to its fluctuation, including the intravascular fluid status, infections and drug interactions. The creatinine was measured immediately upon admission to the emergency room and some patients had received intravenous volume substitution prior to their arrival by the emergency rescue services, causing, probably, some bias to the renal function assessment due to dilution.

## Conclusion

The inappropriate dose reduction of the oral factor Xa inhibitors is associated with lower functional plasma levels, higher clinical stroke severity and worse functional outcome. The high rate of patients with inappropriate regimens warrants additional efforts to confine off-label dosing. Instead of switching to inappropriate low doses, the measurement of the calibrated anti-factor Xa activity could help to improve the safety of anticoagulation treatment in the clinical scenarios of high bleeding risk.
